# Efficient, Automatic, and Reproducible Patch Clamp Data Analysis with “Auto ANT”, a User-Friendly Interface for Batch Analysis of Patch Clamp Recordings

**DOI:** 10.1007/s12021-025-09721-w

**Published:** 2025-03-18

**Authors:** Giusy Pizzirusso, Simon Sundström, Luis Enrique Arroyo-García

**Affiliations:** 1 Department of Neurobiology, Care Sciences and Society, Division of Neurogeriatrics, Karolinska Institutet, 17177 Solna, Sweden; 2 Department of Women’S and Children’S Health, Karolinska Institutet, 17177 Solna, Sweden

**Keywords:** Patch-clamp, Analysis, Automation, Intrinsic properties, Action Potential

## Abstract

Patch-clamp recordings are vital for investigating the electrical properties of excitable cells, yet the analysis of these recordings often involves time-consuming manual procedures prone to variability. To address this challenge, we developed the Auto ANT (Automated Analysis and Tables) open-source software, an automated, user-friendly graphical interface for the extraction of firing properties and passive membrane properties from patch-clamp recordings. Thanks to the novel built-in automation feature, Auto ANT enables batch analysis of multiple files recorded with the same protocol in minutes. Our tool is designed to streamline data analysis, providing a fast, efficient, and reproducible alternative to manual methods. With a focus on accessibility, Auto ANT allows the users to perform precise comprehensive electrophysiological analyses without requiring programming expertise. By combining automation with a user-centric design, Auto ANT offers a valuable resource for researchers to accelerate data analysis while promoting consistency and reproducibility across different studies.

**Supplementary Information:**

The online version contains supplementary material available at 10.1007/s12021-025-09721-w.

## Introduction

Electrophysiological recordings, particularly patch-clamp techniques, are fundamental for understanding the electrical properties of neurons and other excitable cells (Balleza-Tapia et al., 2021; Dallas & Bell, 2021). These techniques allow for precise measurements of action potential waveforms and intrinsic membrane properties, which are crucial for studying neural functionality in health and disease (Lovisolo, 2022). However, the analysis of patch-clamp recordings often remains labour-intensive, requiring manual extraction of key parameters such as spike properties and membrane features (n.d.a). This manual process not only demands expertise and time but also introduces potential variability between analysts.

With the increasing volume of data generated in modern electrophysiology experiments, the need for automated and standardized analysis methods has become evident (Dmitry Tebaykin et al., 2018). Existing electrophysiology tools frequently require extensive programming knowledge (Denker et al., 2018; Rössert & Werner, 2020), or come as online only (Bologna et al., 2021) or non-open source (n.d.b), comprehensive software suites aimed at managing multiple aspects of the experimental workflow (Ma et al., 2024; Zimmermann et al., 2024) —from data acquisition to analysis and statistics—requiring substantial workflow adjustments. This can present a barrier for researchers who prefer to maintain their existing processes or who lack programming experience.

Here, we introduce a solution to these challenges: Auto ANT (Automatic Analysis and Tables), a graphical user interface tool designed for the automated extraction of firing properties and passive membrane properties from multi-sweep patch clamp recordings. Designed with accessibility in mind, Auto ANT allows users with no programming experience to analyse electrophysiological data efficiently and reproducibly, offering a streamlined automation feature for batch analysis of multiple files recorded with the same protocol in just a few steps. By automating the analysis process, Auto ANT minimizes the time required to obtain key electrophysiological measurements while ensuring consistency across experiments, without demanding any major workflow changes.

With Auto ANT, we provide an accessible resource that seamlessly integrates into any electrophysiology workflow, offering high-quality, reproducible data analysis for researchers regardless of programming expertise.

## Materials and Methods

### Software Overview

Auto ANT is a user-friendly graphical interface designed for the automated extraction of passive membrane parameters, firing properties, and action potential waveform analysis from multi-sweep patch-clamp recordings. Auto ANT relies on two well-established feature extraction packages in Python: *eFEL* (Electrophysiology Feature Extraction Library) (Ranjan & Van Geit, 2020) and *IPFX* (Intrinsic Physiology Feature Extractor). These packages are widely used for extracting electrophysiological features from raw recordings, but they require knowledge of the Python programming language, and they do not offer built-in batch analysis solutions for large datasets.

Auto ANT is a complete, standalone application that integrates these tools into an automated and user-friendly interface.The key innovation of Auto ANT lies in its automation feature, which allows users to batch-analyse multiple files recorded with the same protocol in just a few steps. This significantly reduces the time and effort required to analyse large datasets while promoting consistency and reproducibility.

Auto ANT relies on multiple Python packages, with the majority of packages being part of the Python Standard library. These packages include:

*Tkinter,* which is the primary package upon which Auto ANT is built, and *idelib* is used to enable tooltips within the application.

*Sys* and *OS* support accessing local files. The *Threading* package enables multi-threaded processes in Auto ANT, and it is supported by the *Signal* package which is used to terminate active threads when Auto ANT is shut down. Additionally, *Logging* is used to control the logging output of Auto ANT. The *webbrowser* package is used to enable a user to click a button that opens the user’s standard browser and takes them to the published paper, or the GitHub page where Auto ANT is available for download and more usage information is stored. Finally, *Pyinstaller* was used to package the software into an application.

### Input Data Format and Acquisition Mode

**Fig. 1:**
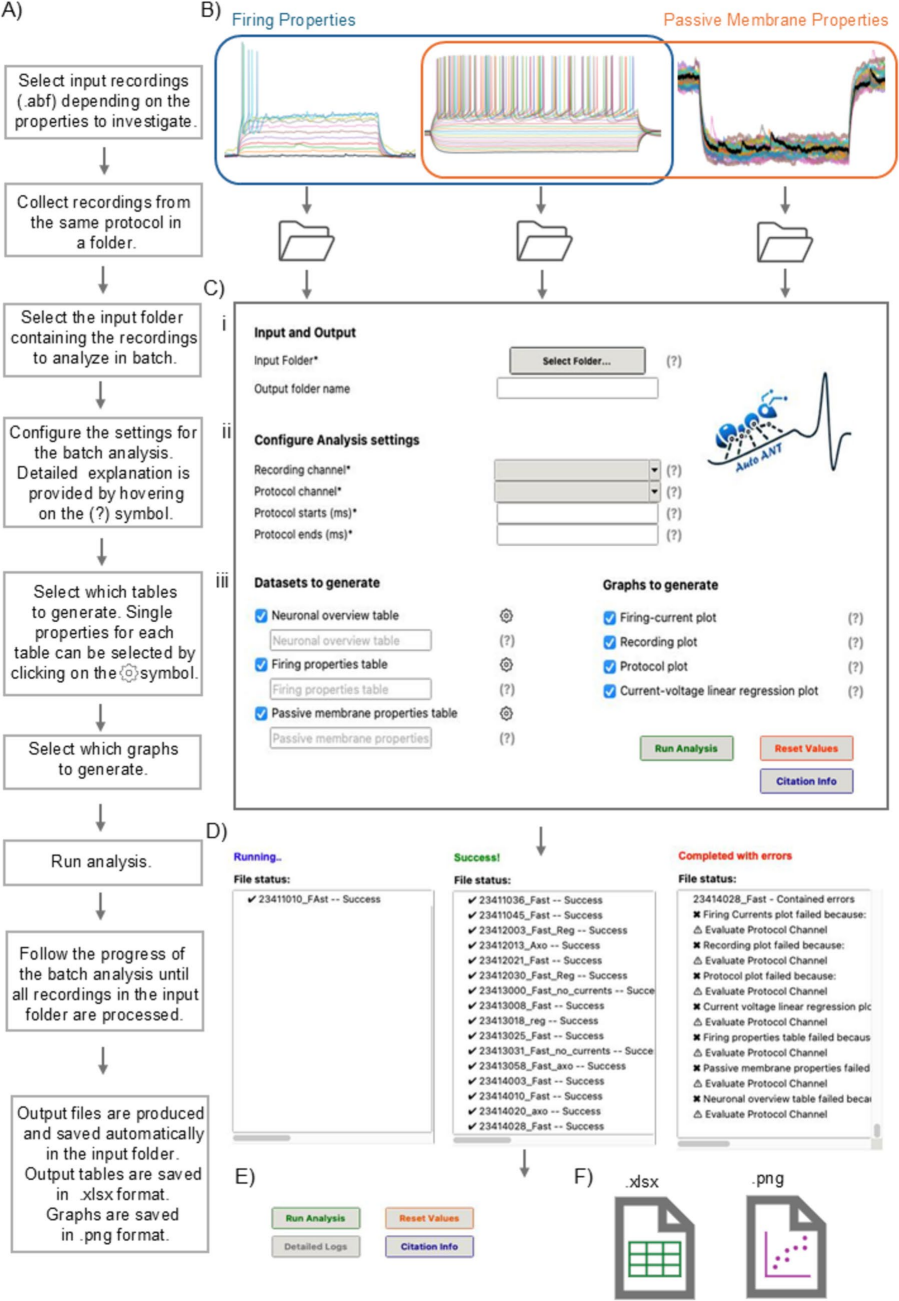
Auto ANT is a user-friendly interface for automated analysis of patch clamp recordings. **A** Workflow diagram with instructions on how to use Auto ANT. **B** Representative recordings that can be analysed with Auto ANT. Recordings acquired with the same protocol are placed in the same folder for batch analysis. **C** Auto ANT window upon launching. From this window the user sets the input and output folder (i), configures the analysis (ii), and selects which tables and graphs to generate (iii) based on the input recordings and analytical needs. **D** Analysis window that appears upon pressing the “run analysis” button, after configuring the analysis. This window shows the analysis progress while the analysis is ongoing (right), and whether the analysis was completed successfully (middle) or with errors (left). **E** When the run is complete, the “detailed logs” button appears, and logs can be accessed. **F** Auto ANT generates the selected tables and graphs that are automatically saved in Excel and PNG format, respectively

**Fig. 2:**
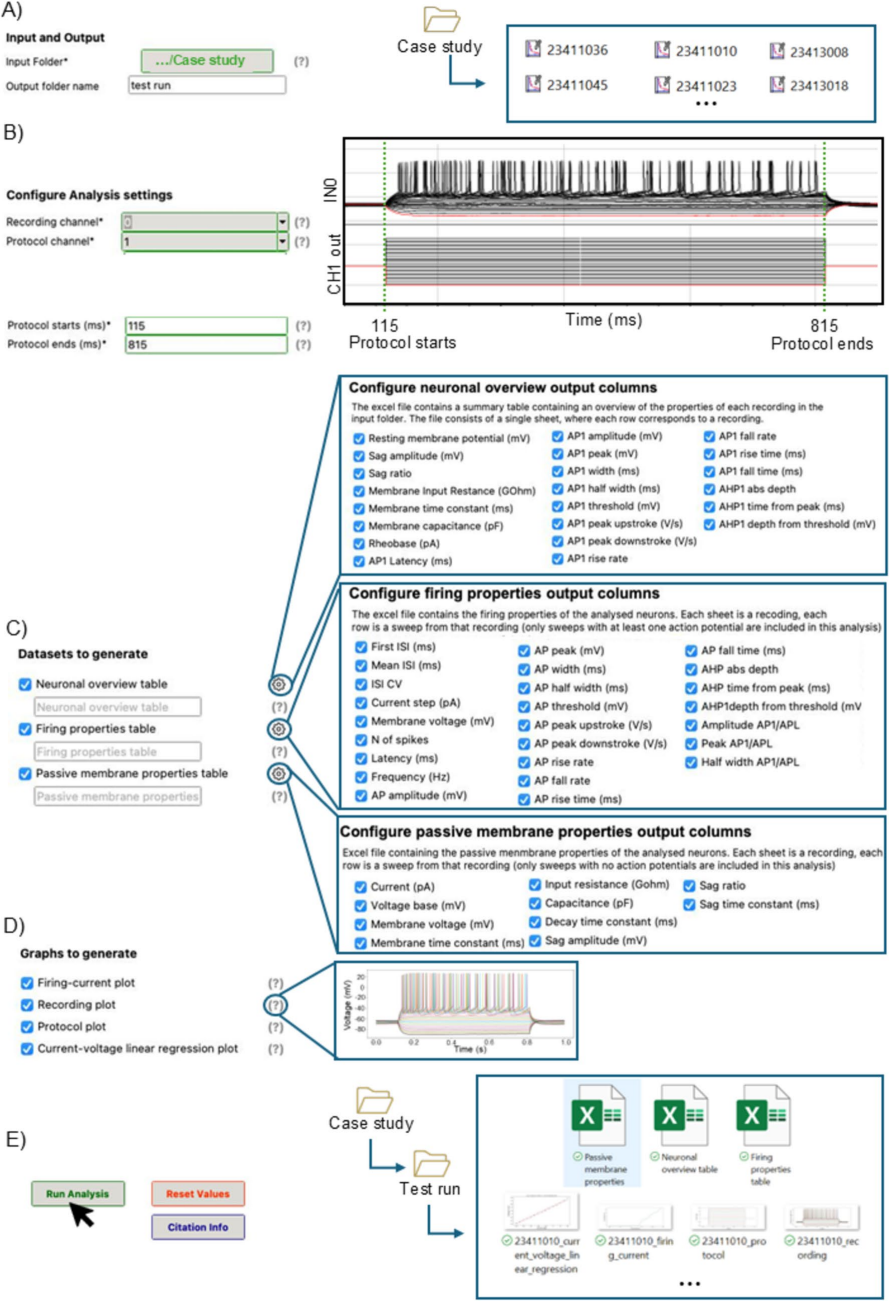
Auto ANT is easy to configure, and it guarantees maximum flexibility by allowing the user to custom which tables, properties and graphs to include in each analysis. **A** The folder containing the recordings for the comparative analysis is set as *Input folder* and the *Output folder* name is set to “Test run”. **B** The analysis settings are configured based on the recordings in the Input folder. On the right, the representative recording file showing where to check *Recording channel*, *Protocol channel*, *Protocol starts* and *Protocol ends* in the .abf file. **C** All tables are selected for this comparative analysis. By clicking on the *gear symbol* adjacent to each table name, the configuration window for the respective table appears. The single properties to include in each table are listed here: all properties are selected for the comparative analysis. **D** All graphs are selected for this comparative analysis. When hovering on the question mark symbol adjacent to each setting, a sample graph or explanation appears. **E** When clicking on the *Run Analysis* button, the butch analysis starts: all recordings contained in the *Input Folder* are analysed and a folder named after the *Output folder name* is automatically created in the *Input Folder*. All the selected outputs are automatically saved in the Output folder

Auto ANT extracts electrophysiological features from recordings obtained in whole-cell patch-clamp mode, specifically supporting files saved in the .abf format (Molecular Devices). The tool accepts multi-sweep recordings from various protocols recorded in current clamp mode, accommodating input recordings with diverse numbers, durations and amplitudes of depolarizing or hyperpolarizing current steps (Fig. 1B). To perform the automated batch analysis, recordings acquired with the same protocol must be placed in a folder and each recording must have a unique name (Fig. 2A). It is important that recordings placed in the same folder have the same protocol duration, while the number and amplitude of steps are irrelevant. For the analysis presented in this paper, we used a protocol of 20 sweeps with a square pulse of 10 pA, from −100 pA to + 100 pA, for 700 ms (Fig. 2B).

### User Instructions

Auto ANT is downloadable at https://github.com/Auto-ANT/Auto-ANT. Instructions for downloading the software are provided (Supplementary material). Upon launching, the interface opens, and the user can configure the analysis (Fig. 1A, C).

*Input and Output* (Fig. 1Ci).

1. *Input Folder*—Folder containing recordings acquired with the same protocol.
2. *Enter output folder name*—The user can select the name of the output folder, which is automatically created inside the Input folder. The outputs of the automated analysis are saved here. If no name is selected, a standard name is assigned to the folder.

*Configure Analysis* (Fig. 1Cii).

1. *Recording channel*—Channel where the recording is stored
2. *Protocol channel*—Channel where the protocol is stored
3. *Protocol starts (ms)*—Time when the current steps start
4. *Protocol ends (ms)—*Time when the current steps end

*Datasets to generate* (Fig. 1Ciii).

The user selects which features to extract from the recordings in the input folder. Firing properties or passive membrane properties can be extracted together or separately, depending on the user’s needs and the specific recordings that are analysed (Fig. 1B). The output tables are automatically saved in Excel format in the user-defined output folder (Fig. 1E). The names of the Excel files for the firing properties and passive membrane properties are selected by the user. If no name is entered, standard names are used.

1. *Firing properties table* – It generates a table containing firing features and single action potential (AP) properties extracted from each sweep of the recordings contained in the input folder. The extraction of AP features requires input recordings performed in current clamp, where at least one depolarizing current step is injected in the cell to induce at least 1 AP (Fig. 1B, blue square). The user selects the intrinsic properties to extract depending on their analytical needs (Fig. 2C). The average properties of all the action potentials on each sweep are calculated (Average AP). Single AP properties are provided for the first, second, second last and last AP (respectively AP1, AP2, APsL and APL) on each sweep.
2. *Passive membrane properties table* – It generates a table containing the passive membrane properties extracted from each sweep of the recordings contained in the input folder. The extraction of passive membrane properties requires input recordings performed in current clamp, where at least one hyperpolarizing current step is injected in the cell (Fig. 1B, orange square). The user selects the intrinsic properties to extract depending on their analytical needs (Fig. 2C).
3. *Neuron overview table –* It generates a summary table containing an overview of the properties of each recording in the input folder. Data in this table are a summary of firing properties and passive membrane properties per neuron, therefore single sweeps are not included in this table. The extraction of the complete set of properties in this table requires input recordings performed in current clamp, where hyperpolarizing and depolarizing current steps are injected into the cell and at least one AP is induced (Fig. 1B, overlap of blue square and orange square). However, the user selects the intrinsic properties to extract depending on their analytical needs (Fig. 2C), hence this table can be adapted to different recording types.

*Graphs to generate* (Fig. 1Ciii).

Similarly, the user selects which graph to generate from the input data (Fig. 2D).

**Fig. 3:**
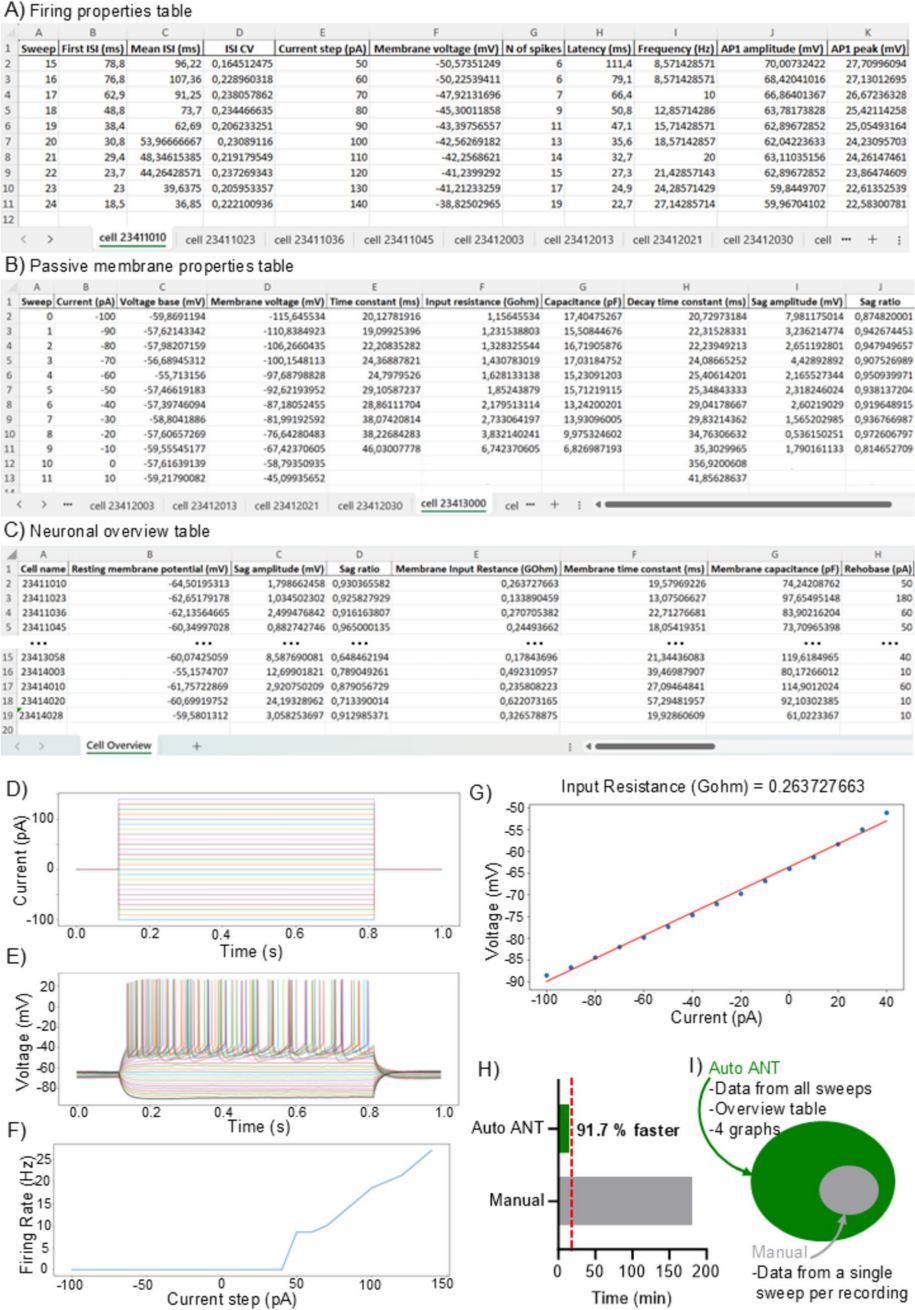
Auto ANT reduces analysis time by 91% and provides more comprehensive datasets when compared to manual analysis. A-C Output tables generated with Auto ANT batch analysis containing information from all recordings in the input folder. Tables are divided into **A** Firing properties table, **B** Passive membrane properties table, and **C** Neuronal overview table. **D**-**G** Output graphs generated with Auto ANT: **D** Protocol plot, **E** Recording plot, **F** Firing-current plot, and **G** Current–voltage linear regression plot. **H** Bar plot showing the time need for Auto ANT batch analysis of all the recordings contained in the input folder versus the estimate time needed for analyzing the same recordings manually. **I** Representative comparison between the amount of data extracted with Auto ANT batch analysis in 15 min and 20 s, versus the amount of data obtained by manual analysis in 180 min

1. Protocol plot – It plots the protocols used for the recording showing the hyper-/de-polarizing current steps delivered to the cell in different colors (Fig. 3D).
2. Recording plot – It plots the recordings contained in the input folder showing the sweeps is different colors, matching the corresponding current step in the protocol plot (Fig. 3E).
3. Firing-current plot – It indicates how much current is needed to make the cell fire at the corresponding firing rate (Fig. 3F).
4. Current–voltage linear regression plot – It indicates how much current is needed to bring the cell to the corresponding voltage. A linear regression model is used to estimate the input resistance of the cell (Fig. 3G). Sweeps containing APs are automatically excluded from this plot.

Once all the configuration for the automated analysis is done, the user presses the “Run analysis” button (Fig. 1C). A window appears indicating the progression and state of the analysis (Fig. 1D). If errors occur, a short description of the error is reported in this window (Fig. 1D). After a run is completed, the "Detailed Logs" button becomes visible (Fig. 1E). Clicking this button opens a new window with the logs from recent runs since the application was opened. The log shows general information about the files being created (tagged as Info), as well as warnings (tagged as Warning) and detailed information regarding errors (tagged as Errors). Errors affecting all recordings in the dataset result in no output table or plot being produced. If an error interests only some recordings in the dataset, those specific recordings will be indicated in the status window and excluded from the output tables, while the rest of the dataset is analysed as usual. If an error affects only a specific output table or plot, only that specific table or plot will be blocked, while all other outputs will be produced normally. A list of common errors is provided in the Supplementary material (2. Error Handling).

### Output Data

General data contained in both *Firing properties* and *Passive membrane properties* tables.

- Sweep – Sweep number (starting from 0).
- Current step (pA) – amount of current injected.
- Steady membrane voltage (mV) – membrane voltage in response to the injected current. It is calculated as the average voltage for the last 10% of the current injection duration.

Firing properties table.

1. Firing features

- N of spikes – number of action potentials (AP) that are detected during the current injection. If spikes happen before or after the current injection, they are automatically excluded.
- Latency (ms) – Time from the start of the current injection to the peak of the first AP.
- Frequency (Hz) – Firing frequency calculated as N of spikes/Current injection duration (ms) * 1000.
- First ISI (ms) – Inter Spike Interval between first and second AP.
- Mean ISI (ms) – Average ISI of all the APs of the sweep.
- ISI CV – Coefficient of variation (CV) of the ISI, calculated as ISI standard deviation/Mean ISI. It is a measure of rhythmicity.

2- Action potential shape features

- AP amplitude (mV) – Height of the AP from firing threshold to peak.
- AP peak (mV) – Maximum voltage reached by the AP.
- AP width (ms) – Width of the AP at the firing threshold.
- AP half width (ms) – Width of the AP at half the AP amplitude.
- AP threshold (mV) – Voltage at the AP start.
- AP peak upstroke (V/s) – Maximum rise rate of the AP (positive number).
- AP peak downstroke (V/s)—Minimum fall rate of the AP (negative number).
- AP rise/fall rate – Rate of change of voltage over time, calculated as dV/dt from AP threshold to AP peak (rise) and AP peak to AHP voltage (fall).
- AP rise/fall time (ms) – Time between AP threshold and peak in the depolarization phase (rise) and repolarization phase (fall) of the AP.
- AHP abs depth – Minimum voltage reached during after hyperpolarization (AHP)
- AHP time from peak (ms) – Time from AP peak to AHP.
- AHP depth from threshold (mV) – Voltage delta between AP threshold and AHP.
- Amplitude AP1/APL – Ratio between the amplitude of the first and the last action potential.
- Peak AP1/APL—Ratio between the peak of the first and the last action potential.
- Half width AP1/APL—Ratio between the half width of the first and the last action potential.

Passive membrane properties table.

- Voltage base (mV) – Membrane voltage before current is injected. It is calculated as the average voltage during the last 10% of the time before the current injection.
- Time constant (ms) – The time it takes for the cell membrane's potential to fall to 63% of its final value after a negative current pulse. It is calculated as the exponential fit of the membrane voltage during hyperpolarization.
- Input resistance (Gohm) – It indicates how much the voltage changes in response to a steady current. It is calculated using Ohm's law as the Steady membrane voltage/current step per sweep.
- Capacitance (pF)—The electrical capacitance of the membrane per sweep calculated as Time constant/Input resistance.
- Decay time constant (ms) – Time constant of the voltage decay after the injected current, calculated as specified for Time constant.
- Sag amplitude (mV) – Amplitude of the sag, calculated as the difference between the minimum membrane voltage reached during hyperpolarization and the steady membrane voltage in the last 10% of the protocol step duration.
- Sag ratio – Ratio between the Voltage delta and the Sag amplitude, calculated as (Voltage base – steady membrane voltage)/Sag amplitude.
- Sag time constant (ms) – Time constant of the exponential voltage decay from the bottom of the sag to the steady membrane voltage.

Neuronal overview table.

- Resting membrane potential (mV) – Membrane voltage when the neuron is in a resting state. It is calculated as the average voltage during the last 10% of the time before the first current step of the protocol is delivered to the neuron.
- Sag amplitude (mV) – Amplitude of the sag, calculated on the most hyperpolarized step of the protocol.
- Sag ratio – Ratio between the Voltage delta and the Sag amplitude calculated on the most hyperpolarized step of the protocol.
- Membrane input resistance (Gohm) – It is a measure of excitability, and it is calculated as the slope of the linear regression of the membrane voltage on the injected current, in absence of AP. If AP are present in the recording, the corresponding sweeps are automatically excluded from this equation.
- Membrane time constant (ms) –It is calculated as the average exponential decay of the membrane voltage in response to the injection of hyperpolarizing current steps between −50pA and −40pA, in absence of AP. If AP are present in the recording, the corresponding sweeps are automatically excluded from this equation.
- Membrane capacitance (pF) – The electrical capacitance of the cell membrane calculated as Membrane time constant/membrane input resistance.
- Rheobase (pA) – It is the amount of current necessary to induce the first AP.
- AP1 intrinsic properties – All the AP waveform features of the first AP fired by the neuron (AP1) are included in this table, as described in the *Firing properties table*.

### Output Data Format

The output generated by Auto ANT consists of ready to use Excel tables: *Firing properties table*, *Passive membrane properties table*, and *Neuronal overview table* (Fig. 3A-C). Users have the flexibility to select which specific variables to include in each table based on their analytical needs (Fig. 2C).

For the *Firing properties table* and the *Passive membrane properties table*, within each Excel file, the name of each sheet corresponds to the name of the respective input recording. Each row in the sheet corresponds to an individual sweep from that recording (Fig. 3A-B).

In the AP properties table, only sweeps that contain at least one action potential are included. Conversely, the passive membrane properties table comprises only those sweeps that do not contain any action potentials. This separation of data ensures that users can efficiently analyse the relevant characteristics of each type of recording.

For the *Neuronal overview table*, the Excel file consists of a single sheet where each row corresponds to a recording (Fig. 3C).

Additionally, Auto ANT provides graphical outputs in the form of plots, which are saved in **.**png format in the same output folder. These graphs can be generated independently or simultaneously with the tables and are automatically named after the input recording, allowing for easy matching with the corresponding sheet in the Excel file and origin cell.

### Comparative Analysis and Data Validation

To validate the accuracy of Auto ANT, we used 18 recordings from a previously published dataset (Pizzirusso et al., 2024). Data acquisition was conducted as detailed in the original paper (Pizzirusso et al., 2024). We placed the 18 recordings, which were obtained using the same current protocol, in a folder (Fig. 2A). All recordings in the folder (Input folder) are on channel 0 (Fig. 2B, Recording channel) while the corresponding current protocols are on channel 1 (Fig. 2B, Protocol channel). The protocols consist of 20 hyper-/de-polarizing current steps of 10 pA, from −100 pA to + 100 pA) delivered to the cell between millisecond 115 (Fig. 2B, Protocol starts) and millisecond 815 (Fig. 2B, Protocol ends). Since the recordings in this dataset contain both hyperpolarizing and depolarizing current steps, we configured Auto ANT to generate the *Firing properties table*, the *Passive membrane properties table* and the *Neuronal overview table* at the same time, along with the four graphical outputs. For the tables, all available properties were selected: 24 firing properties, 10 passive membrane properties and 22 overview properties (Fig. 2C). Then, we compared these properties extracted with Auto ANT with those previously published for the same dataset. For this comparison, we performed a Pearson correlation test, a Bland–Altman analysis, and a paired t-test. Normality was assessed through a Shapiro–Wilk test. All statistical analyses were performed using GraphPad 10.0.2.

### Software Availability

Auto ANT is an open-source software, and it can be downloaded at https://github.com/Auto-ANT/Auto-ANT. The Auto ANT software is available as an easily downloaded application which is compatible with PC and Mac. While Auto ANT compatibility with Linux has not been formally tested, external feedback indicates that it can run on Linux-based systems. Downloading the Auto ANT application requires no programming expertise as it can be launched without any installation process. The raw code is also available. Expert users can modify and adapt the code for specific needs.

## Results

### Auto ANT Provides a Significant Reduction in the Analysis Time When Compared to Manual Analysis

To test Auto ANT, we used 18 recordings from a previously published dataset (Pizzirusso et al., 2024). Auto ANT successfully extracted 34 properties from each sweep of the 18 recordings generating two tables (Fig. 3A-B) with data from all sweeps of each recording, a summary table containing 22 properties per recording (Fig. 3C), and four graphs (Fig. 3D-G) per recording in 15 min and 20 s maximum. The processing time depends on the specifics of the computer used: 15 min and 20 s was the longest processing time (Tested on ZenBook, 2.8 GHz 11th Gen Intel® core™ i7, Windows 11), while 2 min and 5 s was the shortest processing time (Tested on MacBook Pro, 2.3 GHz Quad-Core Intel® core™ i5, MacOS 15.3.1) that we obtained in our tests. In comparison, performing the same analysis manually would require an estimate of 10–15 min to process a single sweep for each recording, depending on the analyst's experience. This time estimate for manual analysis refers to analysis performed using Clampfit (pClamp, Molecular Devices), which is a commonly used software for manual electrophysiology data analysis. In this workflow, spikes are manually detected, and parameters such as amplitude, half-width, and threshold are measured using built-in tools. For passive membrane properties, users measure input resistance, resting membrane potential, and membrane time constant by fitting curves to hyperpolarizing responses (n.d.a). The process must be repeated for each cell and every relevant sweep, leading to a significant time investment and variability between different analysts. Additionally, each measured parameter must be manually written into a spreadsheet, which adds up to the time investment and increases the risk of human error. Auto ANT produced one Excel file for the selected firing properties (*Firing properties table*, Fig. 3A) and one for the selected passive membrane properties (*Passive membrane properties table*, Fig. 3B). Each of these Excel files contains 18 sheets, one per recording, automatically named after the correspondent recording. Each sheet is divided into columns, one per property extracted, and rows, one per sweep of each recording. Sweeps without any action potential are automatically excluded from the Firing properties table. Reversely, only sweeps without action potentials are included in the Passive membrane properties table.

An Excel file for the *Neuronal overview table* is produced too (Fig. 3C). This file provides summary data for each neuron in the dataset, and it contains one single sheet organized in 18 rows, one per recording, with the name of the recording stored in the first column. The following columns of the file correspond to the selected properties for the *Neuronal overview table*.

Additionally, Auto ANT generated four graphs per recording: (1) the protocol plot (Fig. 3D), (2) the recording plot (Fig. 3E), (3) the firing-current plot (Fig. 3F), and (4) the voltage-current plot with a linear regression fit for input resistance calculation (Fig. 3G). These graphs were saved in **.**png format in the output folder, named after the corresponding input recording for easy identification.

In summary, Auto ANT reduced the analysis time for 18 recordings from an estimate of at least 180 min, needed for manual analysis of a single sweep per recording, to a maximum of 15 min and 20 s. Auto ANT not only reduced the analysis time by at least 91.7% (Fig. 3H-I) but also provided additional data for all sweeps in each recording, organised the data from all recordings in a single overview table, and generated ready-to-use graphs, all of which are not included in the manual analysis time estimate.

### Auto ANT Provides Accurate Action Potential and Passive Membrane Properties

To validate the accuracy of the data generated by Auto ANT, we compared the neuronal properties extracted with Auto ANT’s batch analysis with the properties previously published for the same dataset. Published properties for this dataset are: Rheobase, Firing latency, AP amplitude, AP half-width, Peak upstroke, Peak downstroke, Rise rate, Fall rate, Rise time, Resting membrane potential and Input resistance.

To assess comparability, we performed paired t-tests between the properties extracted by Auto ANT, provided in the *Neuronal overview table* (Auto ANT analysis), and those from the published dataset (previous analysis). The t-tests revealed no significant differences for any of the properties, demonstrating that Auto ANT generates results consistent with previous analyses (Table 1, Supp Fig. 1.A_i_-D_i_, Supp Fig. 2.A_i_-D_i_, Supp Fig. 3.A_i_-D_i_). Next, we used a Pearson correlation test and a Bland–Altman test to evaluate the correlation and agreement between the two analyses. The Pearson correlation tests showed a strong positive correlation between the two analyses for all the properties (Table 1, Supp Fig. 1.A_ii_-D_ii_, Supp Fig. 2 .A_ii_-D_ii_, Supp Fig. 3.A_ii_-Di_ii_), indicating that Auto ANT yields similar (Supp Fig. 1.B_ii_-Dii, Supp Fig. 2.B_ii_-D_ii_, Supp Fig. 3.A_ii_-Di_ii_) or identical (Supp Fig. 2.A_ii_, Supp Fig. 3.A_ii_) results to the previous analysis. The Bland–Altman test further confirmed good agreement between the two analyses, with most data points falling within the Limits of Agreement (LoA) intervals (Supp Fig. 1.A_iii_-D_iii_, Supp Fig. 2.A_iii_-D_iii_, Supp Fig. 3.A_iii_-B_iii_). However, there were a few data points approaching the LoA for resting membrane potential (Supp Fig. 3.C_iii_), and input resistance (Supp Fig. 3.D_iii_), indicating that there are some small discrepancies between the Auto ANT analysis and the previous analysis. This can be explained by the different methods used to calculate these properties in the previous analysis and in the Auto ANT analysis.

**Table 1:** Data extracted with Auto ANT is accurate and comparable with the previously published analysis for the same dataset

Auto ANT analysis vs Previous analysis	T-test			Pearson correlation test				Bland Altaman test			
	Mean diff ± SEM	*P* value	Significance	r	95% CI	*P* value	Significance	Bias	SD of bias	95% LoA	Bias in LoA
Rheobase	0,000 ± 12,78	> 0,9999	ns	1	1 to 1	< 0,0001	****	0	0	0 to 0	Yes
Latency	−0,7944 ± 39,88	0.9842	ns	1	1 to 1	< 0,0001	****	0.7944	0.1259	0,5477 to 1,041	Yes
Theshold	1,075 ± 2,216	0.6307	ns	0.988	0,9674 to 0,9956	< 0,0001	****	−1.075	1.154	−3,337 to 1,187	Yes
AP amplitude	−1,075 ± 3,505	0.761	ns	0.9947	0,9855 to 0,9981	< 0,0001	****	1.075	1.154	−1,187 to 3,337	Yes
AP half-width	0,000 ± 0,08456	> 0,9999	ns	1	1 to 1	< 0,0001	****	0	0	0 to 0	Yes
AP peack upstroke	−2,871e-006 ± 12,88	> 0,9999	ns	1	1 to 1	< 0,0001	****	0.000002871	0.00003348	−6,274e-005 to 6,848e-005	Yes
AP peack downstroke	2,344e-005 ± 6,936	> 0,9999	ns	1	1 to 1	< 0,0001	****	−0.00002344	0.00008296	−0,0001860 to 0,0001392	Yes
AP rise rate	3,344 ± 8,637	0.701	ns	0.9904	0,9739 to 0,9965	< 0,0001	****	−3.344	4.212	−11,60 to 4,912	Yes
AP fall rate	−6,289e-006 ± 3,371	> 0,9999	ns	1	1 to 1	< 0,0001	****	0.000006289	0.00003443	−6,119e-005 to 7,377e-005	Yes
AP rise time	−0,05000 ± 0,03303	0.1394	ns	0.8879	0,7192 to 0,9578	< 0,0001	****	0.05	0.05145	−0,05084 to 0,1508	Yes
Resting potential	1,444 ± 1,828	0.4349	ns	0.981	0,9487 to 0,9931	< 0,0001	****	−0.6667	1.088	−2,800 to 1,467	Yes
Input resistance	0,1109 ± 0,07553	0.1512	ns	0.9571	0,8863 to 0,9842	< 0,0001	****	−0.1109	0.1514	−0,4076 to 0,1858	Yes

Table showing the comparison between the Auto ANT analysis and the previously published analysis on the same recordings (*n* = 18). Agreement between the two analyses is evaluated by T-test (right), Pearson correlation test (middle), and Bland–Altman test (left). “Mean diff” is calculated as *Mean Auto ANT – Mean Previous*; “ns” indicates a *p* > 0,05; **** indicates a *p* < 0,0001

The resting membrane potential in the Auto ANT analysis is calculated as the average membrane voltage during the last 10% of the time before the first current step is injected in the cell, while in the previous analysis it is calculated as the average voltage of the entire protocol duration when 0 pA of current is injected in the cell. The input resistance in the Auto ANT analysis is calculated as the slope of the linear regression of the membrane voltage / injected current in the absence of AP, while in the previous analysis it is calculated by averaging the input resistance values from subthreshold hyper- and depolarizing current steps in the input resistance protocol in the absence of AP. Although different methods were used to calculate resting membrane potential and input resistance, the agreement between Auto ANT and previous analysis for these properties was still high (Table 1), as sustained by the fact that no significant difference between Auto ANT and previous analysis was detected by the T-test (Supp Fig. 3.C_i_-D_i_), a significant correlation between Auto ANT and previous analysis is shown in the Pearson test (Supp Fig. 3.C_ii_-D_ii_) and the bias falls in the LoA in the Bland–Altman test (Supp Fig. 3.C_iii_-D_iii_).

Taken together, these results indicate that Auto ANT is a reliable tool for extracting firing properties and passive membrane properties from patch clamp recordings in a standardized, customizable and time-efficient way. Moreover, Auto ANT ensures reproducibility by guaranteeing that identical inputs consistently produce identical outputs, eliminating variability in the analysis process.

## Discussion and Conclusions

Auto ANT is an automated, user-friendly tool designed to streamline the analysis of electrophysiological data by automating the extraction of firing properties and passive membrane properties from multi-sweep patch-clamp recordings. Auto ANT offers significant time efficiency by replacing the manual analysis step, allowing researchers to process large datasets quickly while ensuring standardized, reproducible results.

Although other patch-clamp data analysis tools exist, they are not accessible to users without programming expertise (Denker et al., 2018; n.d.c; Rössert & Werner, 2020) or are designed as more comprehensive software suites (Ma et al., 2024) that require an extensive restructuring of existing workflows, covering data acquisition, analysis, statistics and data storage (Zimmermann et al., 2024). Auto ANT is designed specifically to enhance a single step in the analysis process, removing the programming expertise barrier. Its focus on automated data extraction makes Auto ANT highly versatile and easy to integrate into existing workflows without requiring researchers to adopt an entirely new system. Additionally, Auto ANT is customizable to accommodate specific experimental needs, and its intuitive interface is accessible to users without programming expertise, making it an ideal tool to standardize and accelerate patch-clamp data analysis.

By offering a time-efficient, consistent, and accessible solution for electrophysiology data analysis, Auto ANT meets the needs of researchers who seek to improve workflow efficiency and data quality without disrupting established methodologies.

## Limitations and Future Directions

The current version of Auto ANT is designed to handle .abf files and supports muti-sweep current injection protocols, which are commonly used for patch-clamp analysis. While this ensures broad applicability, it limits compatibility with other file formats and protocol types. Future updates might address these limitations by exploring support for additional data formats and expanding the range of compatible protocols. Additionally, we are committed to maintaining and improving the software by fixing bugs and addressing user-reported issues on GitHub, ensuring its long-term reliability and usability for the electrophysiology community.

## Supplementary Information

Below is the link to the electronic supplementary material.

Supplementary file1 (DOCX 338 KB)

Supplementary file2 (PNG 83 KB)

Supplementary file3 (PNG 87 KB)

Supplementary file4 (PNG 86 KB)

## Data Availability

No datasets were generated or analysed during the current study.

## Abbreviations

AP: Action Potential
AP1: First Action Potential
AP2: Second Action Potential
APsL: Second last Action Potential
APL: Last Action Potential
LoA: Limits of Agreement
