# Supplementary material for: Efficient, Automatic, and Reproducible Patch Clamp Data Analysis with “Auto ANT”, a User-Friendly Interface for Batch Analysis of Patch Clamp Recordings
Source: Neuroinformatics. 2025 Mar 18;23(2):24. doi: 10.1007/s12021-025-09721-w (PMC11920353; doi:10.1007/s12021-025-09721-w)
Supplement: Supplementary file 1 — Supplementary file1 (DOCX 338 KB) [file 12021_2025_9721_MOESM1_ESM.docx]

**Title: Efficient, automatic, and reproducible patch clamp data analysis with “Auto ANT”, a user-friendly interface for batch analysis of patch clamp recordings.**

**Authors:** Giusy Pizzirusso*^#1,2^, Simon Sundström^#1^, Luis Enrique Arroyo-Garcia*^1^

**Affiliations:** ^1^ Department of Neurobiology, Care Sciences and Society, Division of Neurogeriatrics, Karolinska Institutet, 17177 Solna, Sweden

^2^ Department of Women's and Children's Health, Karolinska Institutet, 17177 Solna, Sweden

**Notes:** *Co-corresponding authors

^#^These authors contributed equally

**Supplementary information**

**1. Auto ANT (Automated Analysis and Tables) installation instructions**

Auto ANT can be downloaded in the form of an application or raw code. Both versions require **C++ Build Tools** to be installed on the user’s computer.

- If not already installed, install **C++ Build Tools**

**1.1. Install Auto ANT as an application**

Installing Auto as an application is faster and easier since it does not require any programming expertise or package installation. However, the application versions are heavier (around 90 Mb) than the code version.

To install Auto ANT as an application.

- Download *Auto ANT for PC* or *Auto ANT for Mac* depending on your operating system.
  - Place Auto ANT in your preferred location
- Launch Auto ANT by clicking on the software icon
  - Wait a couple of minutes since the first launching can take longer

**1.2. Install Auto ANT via code**

-
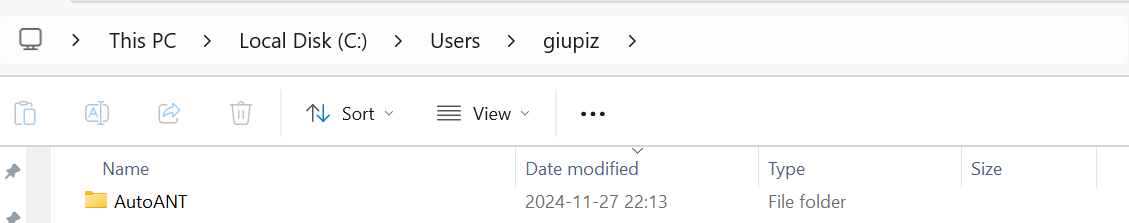
Place the Auto ANT folder in a convenient location (higher up the directory path is better), e.g., in the "users" folder. This will make your folder easy to access later on.
  - For smooth installation, it is important NOT to change any name of the folders and files contained in the Auto ANT folder
  - Unzip AutoANT folder

**Easiest Install: Via Anaconda**

**This has to be done only ONCE**

1. Download and install Anaconda (https://anaconda.org/)

2. In Anaconda Navigator, launch Anaconda_prompt

3. Create a virtual environment (venv)

- In Anaconda Prompt, write "conda create --name environment_name python=3.7.3"
  - Environment_name can be replaced with any name, in this example we called our venv “AutoANTconda”
  -
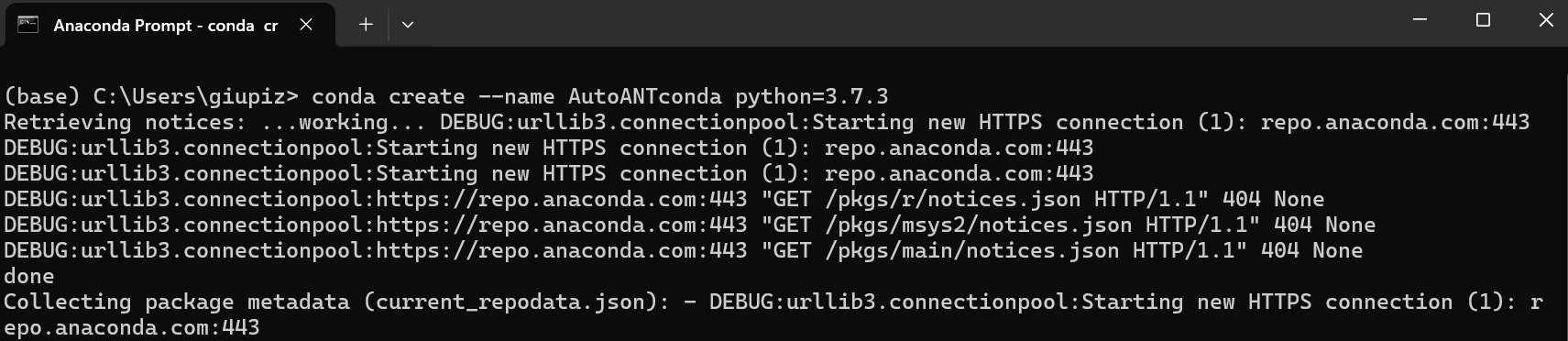
A lot of text will run through the screen
- At some point, it will ask the user if the proposed packages are OK to install, type 'y' and hit enter


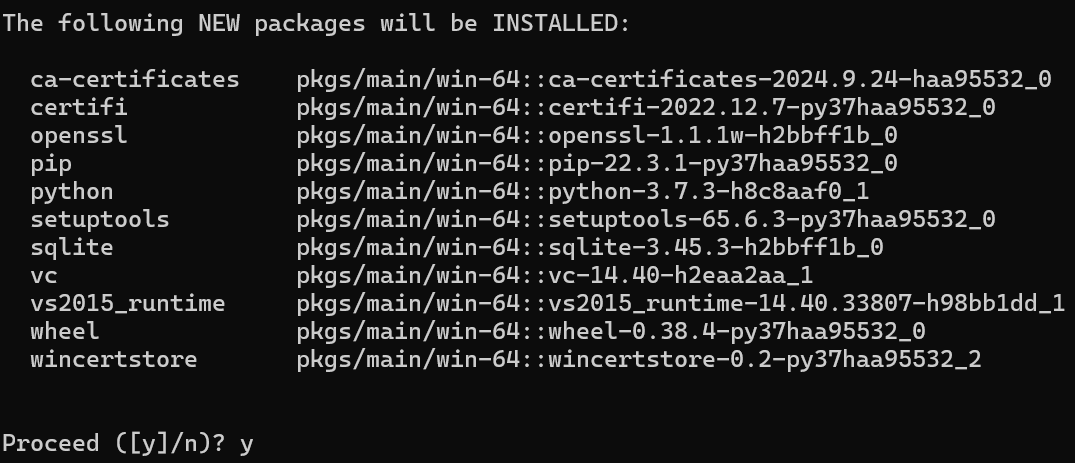


4. Once the venv is created, activate the venv

- In Anaconda Prompt, write "conda activate environment_name "


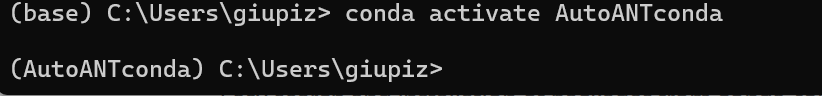


5. Navigate to the AutoANT folder following the folder path

- Use the “cd” command to change directory until you reach the AutoANT folder in the location where you saved it in the beginning


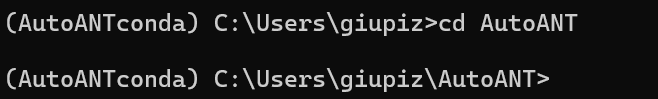


6. Write "pip install -r requirements.txt" - this installs all required packages for Auto ANT (it might take a long time)


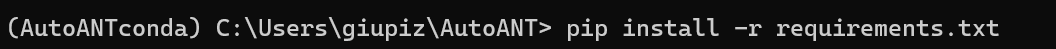


- A lot of text will run through the screen as all packages install.
- If nothing happens with Pip, the environment might be missing Pip. If so, write "conda install pip", and write "y" when prompted.
  - Then, re-run the pip install -r requirements.txt
- If asked to update some packages, type 'y' and hit enter
- In case you get errors, try to google the error you get, and you will most likely find a solution

**1.2.1. How to run Auto ANT via Anaconda (only installation via code)**

**This has to be done every time that you want to launch Auto ANT**

After installing Auto ANT

1. In Anaconda navigator, launch Anaconda prompt

2. In Anaconda prompt, activate the venv

3. Navigate to the AutoANT folder using the “cd” command

4. Write "python run_analysis.py".


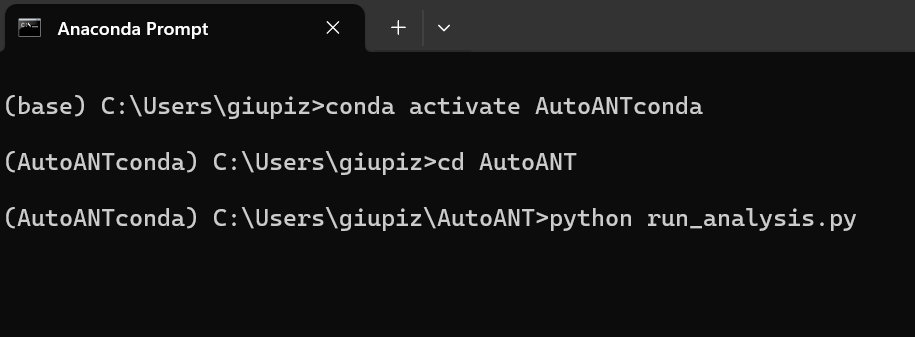


- This will open Auto ANT, which is now ready to help you with your analysis!
  - Launching Auto ANT for the first time will take longer than usual
  - Do NOT close Anaconda prompt while using Auto ANT.


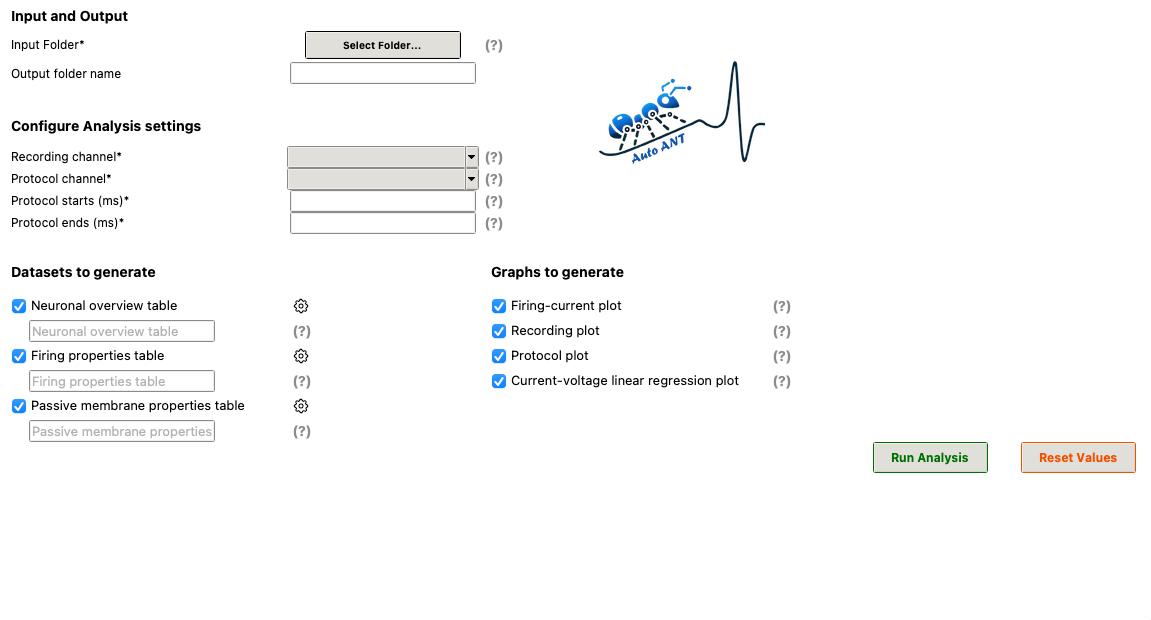

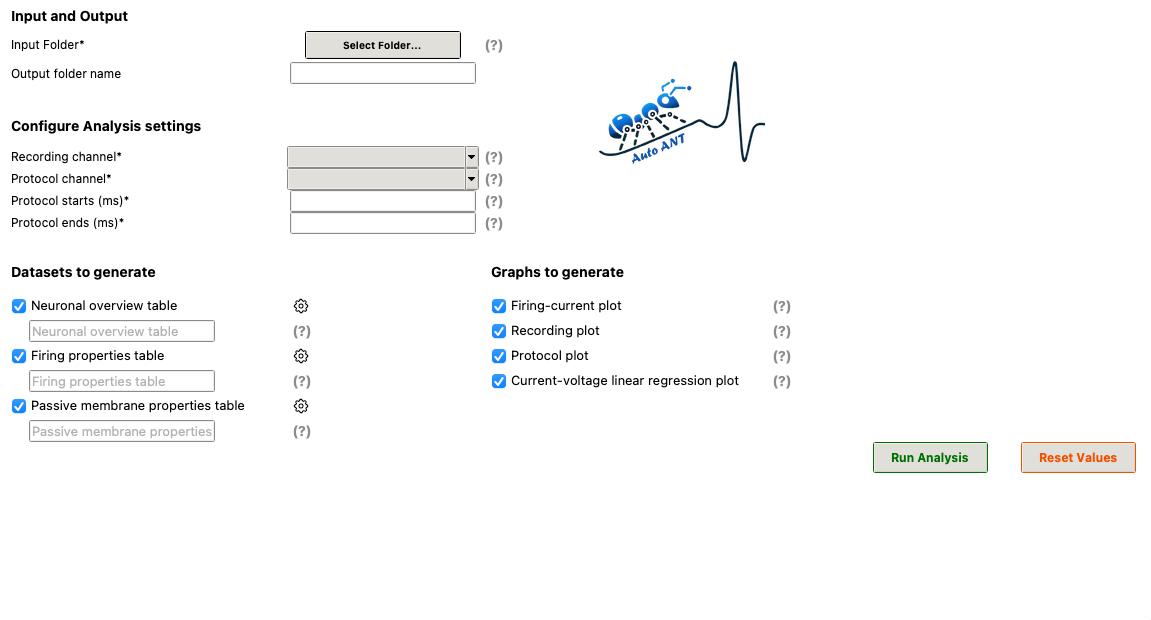

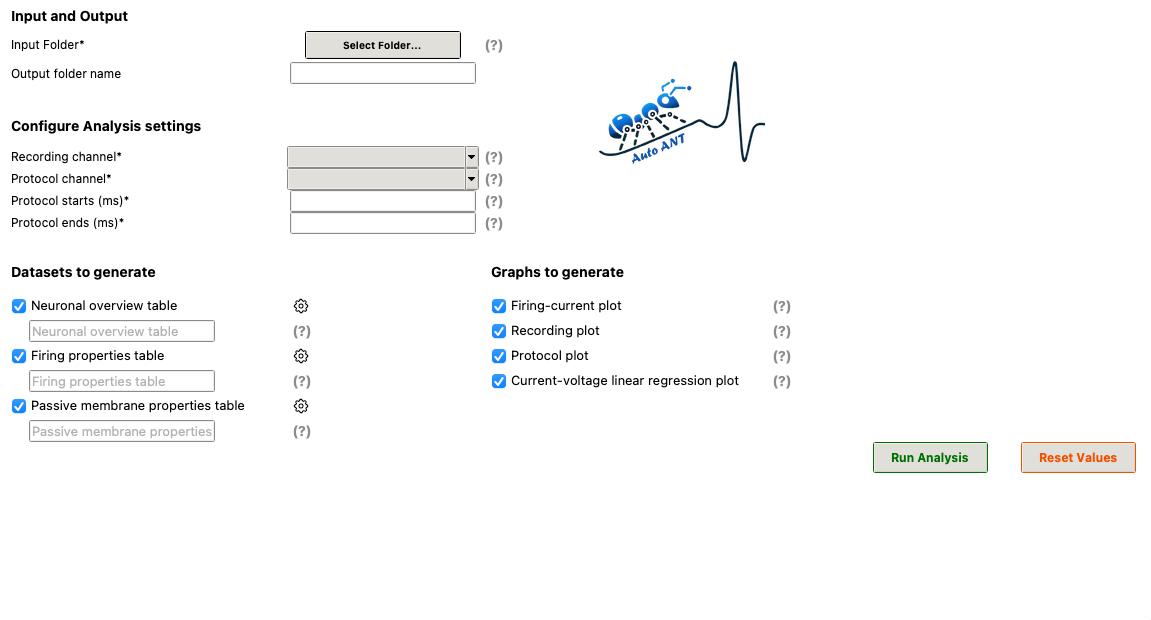

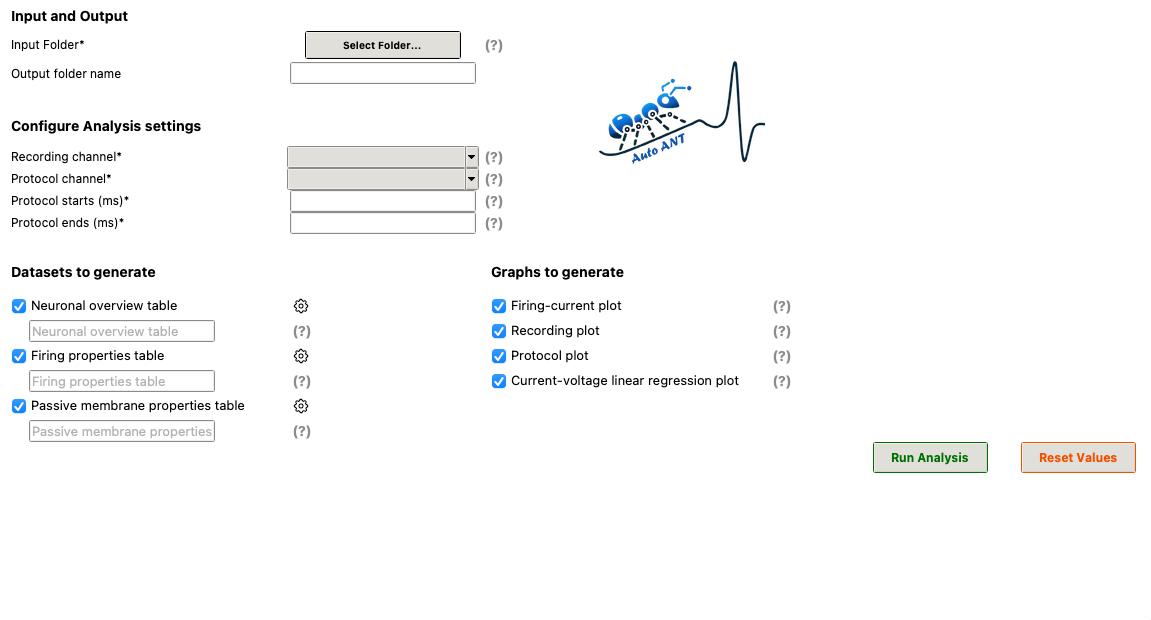


**2. Error handling**

If an error is encountered, a short, summarizing error description is mentioned in the status window. Users can view a more detailed error message within the log.

- If the error affects all recordings in the dataset—such as an incorrect protocol or recording channel—no output will be produced.
- If an error pertains to only some recordings (e.g. a recording acquired with a different protocol from the rest of the dataset), those specific recordings will be indicated in the status window and excluded from the output tables, while the rest of the dataset is analysed as usual.
- If an error affects only a specific output table or plot (e.g., selecting the firing table for recordings without action potentials), only that specific table will be blocked, while all other outputs will function normally.

Common errors to encounter are:

- NoFiringSweepsError
  - Raised when there are no sweeps fulfilling the requirement for firing properties
  - May affect the Firing properties table
- NoNegativeSweepsError
  - Raised when there are no sweeps fulfilling the requirement for membrane
  - May affect the Membrane table, the Neuronal Overview table, or the Current-Voltage linear regression plot
- WrongRecordingChannelError
  - Raised when the wrong recording channel is used
  - May affect all tables and plots
- WrongProtocolChannelError
  - Raised when the wrong protocol channel is used
  - May affect all tables and plots

**3. Supplementary figure legends**

**Supplementary figure 1: Data extracted with Auto ANT are accurate and comparable with the previously published analysis for the same dataset (Part 1)**

**A-D)** Comparisons between **A)** Rheobase, **B)** Firing latency, **C)** AP threshold and **D)** AP amplitude obtained with Auto ANT batch analysis (Auto ANT analysis) and previously published data (Previous analysis). Agreement between current and previous analysis was evaluated with a t-test (i), a Pearson correlation test (ii) and a Bland-Altman test (iii) for each variable.

**Supplementary figure 2: Data extracted with Auto ANT are accurate (Part 2)**

**A-D)** Comparisons between **A)** AP half-width, **B)** AP peak upstroke, **C)** AP peak downstroke and **D)** AP rise rate obtained with Auto ANT batch analysis (Auto ANT analysis) and previously published data (Previous analysis). Agreement between current and previous analysis was evaluated with a t-test (i), a Pearson correlation test (ii) and a Bland-Altman test (iii) for each variable.

**Supplementary figure 3: Data extracted with Auto ANT are accurate (Part 3)**

**A-D)** Comparisons between **A)** AP fall rate, **B)** AP rise time, **C)** Membrane potential and **D)** Input resistance obtained with Auto ANT batch analysis (Auto ANT analysis) and previously published data (Previous analysis). Agreement between current and previous analysis was evaluated with a t-test (i), a Pearson correlation test (ii) and a Bland-Altman test (iii) for each variable.
