# Supplementary figures and images for: Efficient, Automatic, and Reproducible Patch Clamp Data Analysis with “Auto ANT”, a User-Friendly Interface for Batch Analysis of Patch Clamp Recordings
Source: Neuroinformatics. 2025 Mar 18;23(2):24. doi: 10.1007/s12021-025-09721-w (PMC11920353; doi:10.1007/s12021-025-09721-w)

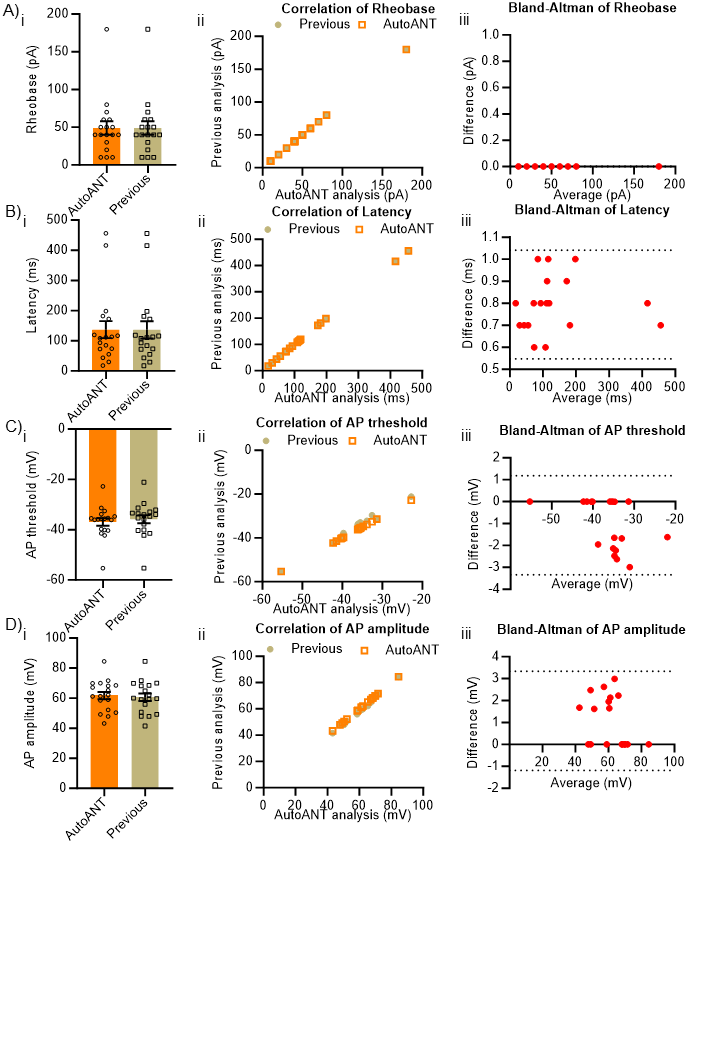

Supplement: Supplementary file 2 — Supplementary file2 (PNG 83 KB) [file 12021_2025_9721_MOESM2_ESM.png]

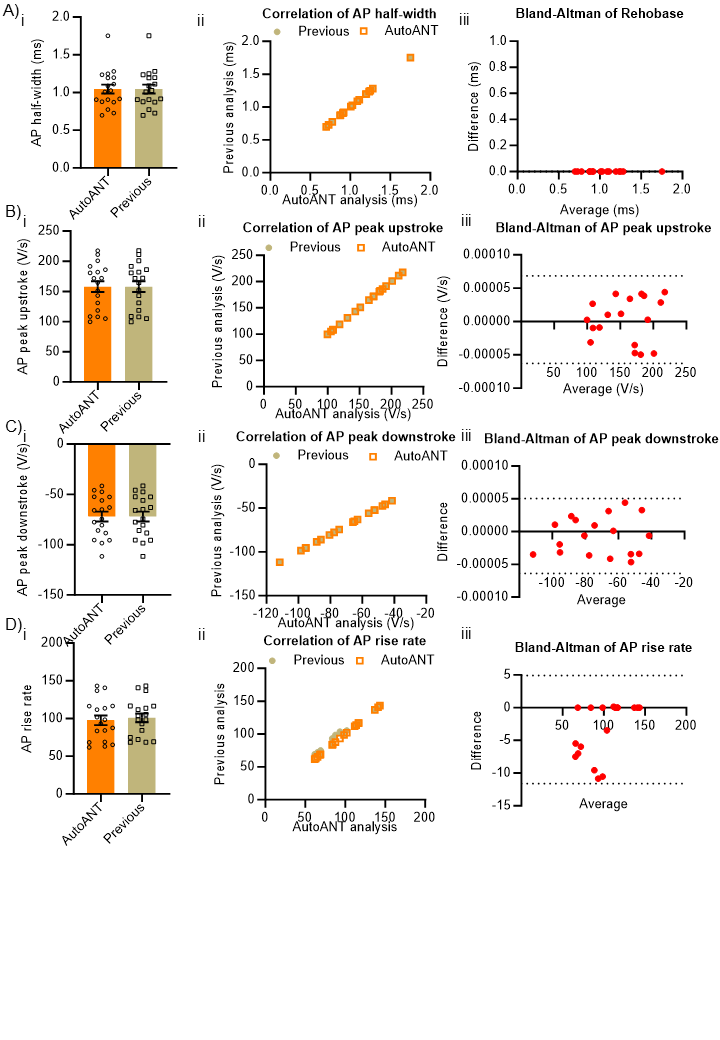

Supplement: Supplementary file 3 — Supplementary file3 (PNG 87 KB) [file 12021_2025_9721_MOESM3_ESM.png]

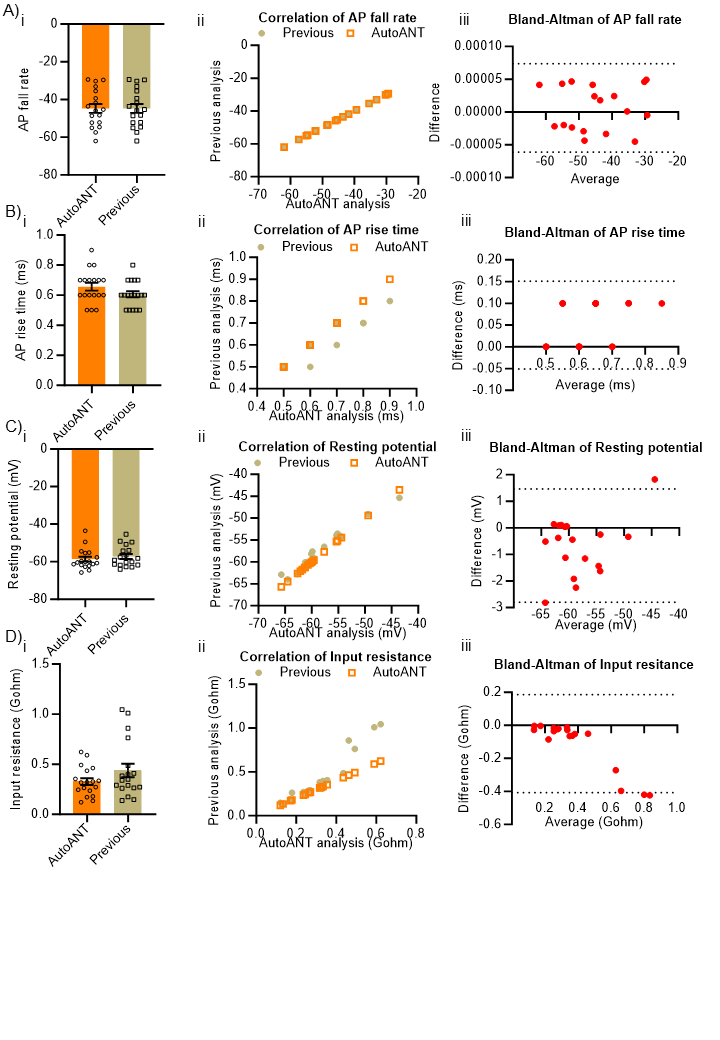

Supplement: Supplementary file 4 — Supplementary file4 (PNG 86 KB) [file 12021_2025_9721_MOESM4_ESM.png]
